# Retraction

**DOI:** 10.1111/1753-0407.13255

**Published:** 2022-03-17

**Retraction:** Mohamed Salih, S., Nallasamy, P., Muniyandi, P., Periyasami, V. and Carani Venkatraman, A. (2009), Genistein improves liver function and attenuates non‐alcoholic fatty liver disease in a rat model of insulin resistance. *Journal of Diabetes*, 1:278–287. https://doi.org/10.1111/j.1753-0407.2009.00045.x.

The above article, first published online on October 27, 2009 in Wiley Online Library (wileyonlinelibrary.com), has been retracted by agreement between the Editors‐in‐Chief, Zachary T. Bloomgarden and Guang Ning, Ruijin Hospital, Shanghai Jiaotong University School of Medicine and John Wiley & Sons Australia, Ltd. The retraction has been agreed as Figure 4 was found to have been published in another article (Suppression of hepatic oxidative events and regulation of eNOS expression in the liver by naringenin in fructose‐administered rats. *Eur J Pharmacol*. 2010 Oct 25;645[1‐3]:177–184) by the same group of authors but purporting to show different results. The authors were unable to provide a satisfactory explanation and did not provide the data used in creating the Figure. Furthermore, the Editors, along with an additional reviewer, have been unable to confirm that Figure 4 supports the results and conclusions of the article. Based on this, the Editors hereby retract the article.

